# Assessing virulence of *Varroa destructor* mites from different honey bee management regimes

**DOI:** 10.1007/s13592-019-00716-6

**Published:** 2019-12-10

**Authors:** Travis L. Dynes, Jennifer A. Berry, Keith S. Delaplane, Jacobus C. de Roode, Berry J. Brosi

**Affiliations:** 1 Department of Environmental Sciences, Emory University, Atlanta, GA 30322 USA; 2 Department of Entomology, University of Georgia, Athens, GA USA; 3 Department of Biology, Emory University, Atlanta, GA USA

**Keywords:** Apis mellifera, growth rate, host management, virulence, Varroa destructor

## Abstract

The mite *Varroa destructor* is an important honey bee parasite that causes substantial losses of honey bee colonies worldwide. Evolutionary theory suggests that the high densities at which honey bees are managed in large-scale beekeeping settings will likely select for mites with greater growth and virulence, thereby potentially explaining the major damage done by these mites. We tested this hypothesis by collecting mites from feral bee colonies, “lightly” managed colonies (those from small-scale sedentary operations), and “heavily” managed colonies (those from large-scale operations that move thousands of colonies across the US on a yearly basis). We established 8 apiaries, each consisting of 11 colonies from a standardized lightly managed bee background that were cleared of mites, and artificially infested each apiary with controlled numbers of mites from feral, lightly managed, or heavily managed bees or left uninoculated as negative control. We monitored the colonies for more than 2 years for mite levels, colony strength (adult bee population, brood coverage, and honey storage), and survival. As predicted by evolutionary theory, we found that colonies inoculated with mites from managed backgrounds had increased *V. destructor* mite levels relative to those with mites from feral colonies or negative controls. However, we did not see a difference between heavily and lightly managed colonies, and these higher mite burdens did not translate into greater virulence, as measured by reductions in colony strength and survival. Our results suggest that human management of honey bee colonies may favor the increased population growth rate of *V. destructor*, but that a range of potential confounders (including viral infections and genotype-by-genotype interactions) likely contribute to the relationship between mite reproduction and virulence.

**Electronic supplementary material:**

The online version of this article (10.1007/s13592-019-00716-6) contains supplementary material, which is available to authorized users.

## Introduction

European honey bee (*Apis mellifera* L.) colonies have experienced widespread losses in the past decades in the US and Europe, which is a particular concern due to the importance that honey bees play in agricultural pollination services critical to both the economy and human health (National Research Council 57; Pettis and Delaplane 59). While honey bees are facing numerous challenges, from pesticides to land use changes, parasites have emerged as a significant factor in these losses (Potts et al. 60). In the first half of the 20th century, the obligate ectoparasitic mite *Varroa destructor* (Acari: Mesostigmata: Varroidae) made a sustained host switch from the Asian honey bee (*Apis cerana*) to the European honey bee (Rosenkranz et al. 65). Since that time, *V. destructor* has spread around the world and become the largest biotic threat, termed “varroosis”, currently facing the beekeeping industry (Sammataro et al. 66; Rosenkranz et al. 65). In addition, *V. destructor* is a vector for a range of economically important viruses, and the interaction between these viruses and *V. destructor* is considered the single most important factor in honey bee colony losses worldwide (Boecking and Genersch 9; Wegener et al. 71).

In the honey bee\ system, the dynamics by which *V. destructor* mites interact with honey bee colonies can vary drastically. Feral honey bee colonies, those colonies that are unmanaged by humans, typically occur at a density of around one per square kilometer in the USA (Seeley 67). In these isolated settings, bees and mites are not likely to interact with individuals from other honey bee colonies on a regular basis. In contrast, industrial beekeeping operations manage thousands of colonies in a much smaller area. Virulence-transmission trade-off theory (Boots and Sasaki 11; Boots et al. 12; Alizon et al. 2; Lion and Boots 50; Webb et al. 70) suggests that the higher colony densities and high rates of between-colony mixing found in managed operations favor *V. destructor* mites with increased reproduction and virulence. According to trade-off theory, natural selection favors virulent parasites that cause reductions in host fitness by selecting for between-host parasite transmission (Levin and Pimentel 49; Anderson and May 5; Ewald 31; Bremermann and Pickering 14; Antia et al. 6; Bull 16; Levin 48; Boots and Mealor 10). This theory is based on the assumption that both between-host transmission and virulence (usually defined as parasite-induced host mortality) increase with increasing within-host parasite reproduction, an assumption that has found empirical support in a wide range of systems (Messenger et al. 54; Mackinnon and Read 51, 52; Jensen et al. 40; De Roode et al. 25; Hawley et al. 37). As a result, parasites are generally expected to evolve an intermediate level of within-host growth and consequent virulence: parasites with low growth rates are selected against because of low between-host transmission, while parasites with high growth rates are selected against by killing the host before transmission can occur (Levin and Pimentel 49; Lenski and May 46). The expected level of optimal virulence, however, depends strongly on the density of susceptible host individuals, as well as the spatial structure of the population (Kamo and Boots 42; Boots and Mealor 10). In well-mixed high-density host populations, transmission opportunities are ample and the cost of high virulence in terms of killing hosts before transmitting is low. This type of environment is common in agricultural settings and according to theory can favor the evolution of higher virulence (Kennedy et al. 43). In contrast, in highly structured low-density host populations, transmission opportunities are rare and costs of virulence are high. As a result, evolutionary theory predicts selection for greater virulence in highly dense and well-mixed populations than in low density populations with high spatial structure. Evidence for such increased virulence evolution due to greater host density remains lacking outside of laboratory settings (Kerr et al. 44; Boots and Mealor 10), but it is now clear that practices imposed by agriculture can select for more deadly parasites, as has been demonstrated, for example, in the increased virulence of the virus causing Marek’s disease due to vaccination of chickens with a vaccine that provides tolerance, but not resistance, to the target virus (Atkins et al. 7; Read et al. 62).

The contrasting transmission conditions driven by density and population mixing are crucial to honey bees, where industrial beekeeping practices have shifted the host-parasite interaction from low densities with high spatial structure in feral bees to highly dense and well-mixed populations in industrially managed bees. Thus, based on virulence-transmission trade-off theory, we would expect greater selection for parasite growth and virulence in managed honey bee colonies than in feral colonies (Brosi et al. 15). By promoting increased transmission opportunities, management practices such as moving frames of brood to boost struggling colonies (a common beekeeping practice) and the high rates of mixing of managed bees due to migratory beekeeping could contribute to *Varroa destructor* virulence evolution and be responsible for maintaining virulent *Varroa destructor* genotypes in managed honey bee colonies (Fries and Camazine 32; Calderón et al. 17; Guzmán-Novoa et al. 36; Brosi et al. 15).

Our current understanding of these relationships in the honey bee system is limited, but there is a small amount of research that is consistent with the virulence-transmission trade-off hypothesis. Based on a comparison of bee colonies infected with mites from different backgrounds, Seeley (67) proposed that avirulent mite strains may explain feral colonies surviving *V. destructor* better than feral bee resistance to the mites. Migratory beekeepers have reported more colony mortality than small-scale beekeepers (Dahle 22). More *V. destructor* transmission has been observed in higher-density (compared to lower-density) honey bee colonies (Nolan and Delaplane 58; Dynes et al. 30). Furthermore, studies indicate a genetic basis for variation in mite virulence, confirming that virulence could be acted upon by natural selection (De Jong and Soares 23; Anderson 4; Corrêa-Marques et al. 20, 21).

To understand if mites from different management regimes have evolved contrasting virulence, we completed a large and replicated study at the apiary level to examine varroosis using a highly standardized approach which to our knowledge has not been previously attempted. Specifically, we compared how mites evolved from different honey bee management histories (feral, lightly managed, or heavily managed) reproduced and affected bee colonies from a common, lightly managed background. We hypothesized that *V. destructor* mites that evolved under more intensive honey bee management regimes had greater population growth rates and increased virulence compared with lower honey bee management intensity. We measured both mite burdens and effects on colony strength over more than 2 years. The strength of our approach lies in our colony and queen standardization, mite clearance, standardized inoculations, and replication at the apiary level.

## Materials and methods

### Overview

We performed a virulence assay on *V. destructor* mites collected from different honey bee management backgrounds on bees obtained from a lightly managed background such as one would find with backyard beekeepers. Our purpose was to determine whether management conditions have selected for mites with differential growth rate and/or virulence and whether colony response differs among these backgrounds. We established eight apiaries, each consisting of 11 colonies, for a total of 88 colonies, in June 2015 around Athens, GA, USA, maintained by the University of Georgia Honey Bee Lab. Colonies were initially cleared of mites and subsequently inoculated with mites (*N* = 100 in multiple doses over the course of 2 months). We used 7–9 mite donor colonies for each management background type (feral, lightly managed, and heavily managed). In order to ensure a sufficient quantity of mite inoculations for each experimental colony, mites were pooled from between 1 and 3 of the 7–9 possible donor colonies (Table I). Colonies in two apiaries each were inoculated with mites from feral, lightly managed, or heavily managed backgrounds, while two apiaries were established as negative controls and were not inoculated with mites.

**Table I:** Mite inoculation sources within each apiary

Apiary	Mite background	Number of colonies receiving mites (mite donor source)
1	Negative control	NA
2	Heavily managed	5 (HM7), 2 (HM1/6), 1 (HM8/13), 1 (HM10/12), 1 (HM6/10/12)
3	Lightly managed	3 (LM1/8), 2 (LM2), 2 (LM3), 2 (LM6/29), 1 (LM5)
4	Feral	4 (F7/13), 2 (F1), 2 (F3/10), 1 (F6), 1 (F2/14), 1 (F6/13)
5	Lightly managed	3 (LM5), 2 (LM2), 2 (LM3), 2 (LM6/Farm9), 1 (LM1/8), 1 (LM1/2/8)
6	Heavily managed	5 (HM7), 2 (HM1/6), 2 (HM10/12), 1 (HM2/27), 1 (HM8/13)
7	Negative control	NA
8	Feral	5 (F7/13), 3 (F6), 1 (F1/2), 1 (F2/14), 1 (F3/F10)

### Mite and honey bee backgrounds

#### Mite sources

We collected live mites from different source backgrounds by dusting colonies with powdered sugar and gathered mites that were dislodged and fell onto a piece of cardboard placed on the bottom of the hive. Mites from feral backgrounds were obtained from honey bee colonies that originated from swarm traps placed in remote forest settings (to reduce likelihood of swarms from recently managed colonies) in Georgia (Oconee National Forest or the Okefenokee National Wildlife Refuge), while mites from lightly managed backgrounds originated from colonies from typical backyard beekeeper management systems. For the heavily managed mites, we acquired mites from a migratory beekeeper that manages thousands of colonies. Colonies were housed in standard five-frame Langstroth nucleus hive boxes and we attempted to minimize drift by arranging colonies in a circular layout with all entrances facing outwards from the center of the circle, with 1 m between the colonies. We further attempted to minimize drift by maximizing bees’ ability to visually distinguish between colonies (Dynes et al. 30). The colonies were painted different colors, placed at different heights above the ground (5, 20, or 40 cm), with different symbols painted at the hive entrance.

#### Colony standardization, mite clearance, and mite inoculation

We started with highly standardized colonies to minimize variation. We obtained mated queens from a single queen breeder in southern Georgia, USA, and added 1.1 kg (2.5 lb) adult bees from a common genetic background to each package. To clear mites from the standardized packages, we placed them in a dark room overnight at 16.6 °C (62 °F) and sprayed with sugar water 1 h prior to the application of 30 mL of a 2.8% oxalic acid solution (Milani 55). Each package was installed 3 days later into a nucleus colony in a randomly assigned apiary at least 5 km from any known colonies (Figure S1, map). Mites were collected from source colonies outside of the experiment by sifting powdered sugar over the colony and collecting dislodged mites at the bottom of the colony. We used small natural fibered paintbrushes to place mites on damp coffee filters. We kept mites in an incubator set at 35 °C (95 °F) until all mites were collected for each dose. We then transferred all mites (*N* = 100 mites per colony) evenly to an uncapped brood frame and waited to ensure that mites were crawling before returning the frame to the colony.

To maintain our focus on these original colonies (and their queens), we enacted swarm control on colonies likely to swarm by splitting those colonies. We standardized swarm control in this manner to ensure that small colonies were not jeopardized by the procedure. A total of 33 out of the 72 colonies that remained alive were split in March and April of 2016. We employed a Fisher’s exact test to determine that there was not a statistically significant difference (*X*^2^(3) = 6.44, *P* = 0.092) in amount of splitting between our treatment groups. During the experiment, we did not conduct any control measures against *V. destructor*. We continued the experiment from June 2015 through December 2017, at which point only 12 of the original 88 colonies were surviving.

### Data collection

#### Measuring *V. destructor* infestation

We measured *V. destructor* infestation levels using three different methods. First, we used an alcohol wash method described by Fries et al. (33). This method involves destructively sampling approximately 300 bees from a colony in alcohol and counting bees and mites (which detach from the bees allowing easier counting) to get a relative mite level on the adult bee population. We took eight alcohol wash samples throughout the experiment (roughly once a month during summer and fall and once every 3 months at other times of the year). Second, we used sticky boards (Branco et al. 13), a standard method to evaluate *V. destructor* levels in a colony by collecting mites that fall and become entrapped on a board placed at the bottom of a colony. We measured mite levels with sticky boards six times throughout the experiment including one measurement immediately following package installation to confirm that colonies were *V. destructor* free (roughly every 3 months during the first year and at the end of the experiment). Third, we measured the mite population in brood cells by opening 100 covered brood cells in each colony and counting the number of mites. We measured mite levels in brood cells five times throughout the experiment (roughly every 4 months).

#### Colony strength assessments

We took periodic strength assessments throughout the experiment in order to evaluate the effect of mite background on colony strength. We followed the assessment guidelines outlined in Delaplane et al. (27) to measure colony strength in terms of (1) adult bee population, (2) amount of brood, and (3) amount of honey stored for each colony. We performed these colony assessments five times over the 2 years of the experiment (roughly every 4 months). We also recorded the date each colony was found to be dead and last known date it was alive for survival analyses.

### Statistical analysis

#### Overview

We explored how our treatment levels (mites from feral, lightly managed, and heavily managed backgrounds) affected the mite burdens and health response outcomes at the colony level. We also assessed the effects of mites from our different mite donor colonies within each treatment level to determine whether variation exists within the treatment levels. We conducted analyses based on three classes of response variables: (1) colony-level mite infestation levels, (2) colony strength parameters, and (3) colony-level survival.

#### Mite infestation levels and colony strength

Our experiment used longitudinal repeated measures and nested random effects which can result in temporal and within-subject autocorrelation and violates the assumption of independence for parametric and linear regression methods. Therefore, we used generalized estimation equations (GEE) to account for repeated measures including temporal autocorrelation. GEE models are similar to the more common generalized linear mixed models (GLMM), but handle within-group correlation as a marginal model rather than as a conditional model found in GLMMs (Hubbard et al. 39). We used the ‘geeglm’ function in the ‘geepack’ package v1.2-1 (Højsgaard et al. 38) in R v.3.4.2 (R Core Team 19) to specify and evaluate the GEE models in particular because it allows for longitudinal data with missing observations. We blocked the data by apiary and colony and utilized an autoregressive (AR1) autocorrelation structure to compare treatment levels with negative control colonies. We used the ‘lsmeans’ package v. 2.27 in R to conduct post hoc pairwise comparisons of response variables of mites from different donor colonies using Tukey’s method for multiple comparisons (Lenth 47). We used the ‘missMDA’ package v.1.12 in R (Josse and Husson 41) to impute missing values (*N* = 917 out of a total of 1869 values) for mite measurements that did not occur in the same months and then created a composite index combining the three methods of mite measure using a unity-based normalization index (Dodge et al. 28). This index takes each method of mite measurement and scales the measurement to a value between 0 and 1 by comparing the measurement to the minimum and maximum value for that method. The normalized value for each method of measurement is then added to the other methods for that particular sample for a composite index value. We employed a GEE model to evaluate this composite index in addition to each of the individual mite measures. We similarly assessed colony strength measures (adult bee population, brood production, and honey stores) using GEE models to compare treatment levels to negative control colonies.

#### Survival analysis

We performed survival analyses to determine whether there was a difference in colony survival based on mite background. Colonies were inspected periodically throughout the experiment and exact timing of colony death could not be determined. Therefore, we used an interval of date of observed colony death and date of last known colony viability. Given this data structure, we analyzed survival with mixed-effects survival (frailty) Cox proportional hazard models, with interval censoring via the ‘frailtypack’ package (Rondeau et al. 64) in R.

## Results

### Overview

We collected data on mite levels and colony strength parameters for each colony. The colony strength assessments resulted in 231 measurements from each colony on the adult bee population, brood coverage, and honey storage. In order to evaluate *V. destructor* levels throughout the experiment, we collected 413 sticky boards, 353 alcohol washes (each containing approximately 300 worker bees), and 189 counts of mites in the brood (each including 100 brood cells).

### Mite infestation levels

**Figure 1.**
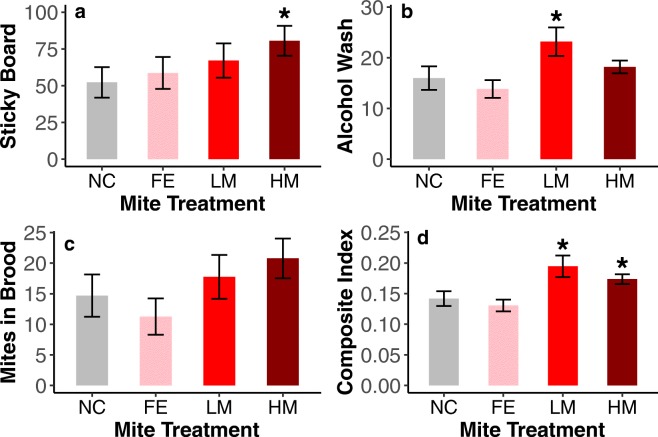
Measures of mite abundance by treatment over the course of the experiment (NC = Negative Control, FE = Feral, LM = Lightly Managed, HM = Heavily Managed). **a** Sticky board, **b** alcohol wash, **c** mites in brood, and **d** composite index of all three measurements. GEE models were employed for data in each panel to determine significant differences from the negative controls. More mites were found in colonies with mites from heavily managed backgrounds (**a** Wald = 4.06, *P* = 0.044) and lightly managed backgrounds (**b** Wald = 3.94, *P* = 0.047). Note that while significance was not always found in each mite measurement (**a**–**c**), the trend in each is consistent with our hypothesis. A unity-based normalization index was used in panel **d** to combine all three mite measurements. This reduced the measurement variation and showed a significant difference between mites from the lightly managed (Wald = 5.99, *P* = 0.014) and heavily managed (Wald = 4.55, *P* = 0.033) backgrounds from the negative controls which is consistent with our hypothesis. Error bars represent SEM.

The GEE model for mite levels as assessed by sticky boards showed that colonies inoculated with mites from heavily managed backgrounds had significantly (Wald = 4.06, *P* = 0.044) higher mite levels over the course of the experiment than the negative control colonies (Figure 1a). The model for the alcohol wash data showed that colonies inoculated with mites from lightly managed backgrounds had significantly (Wald = 3.94, *P* = 0.047) higher mite levels (Figure 1b). The mites in brood measurement did not show any treatment level significantly different from negative controls (Figure 1c). However, the trend in this measurement is consistent with the other two measures with colonies inoculated with feral mites tending to have the lowest mite levels and the treatment groups from managed backgrounds having the most mites. The GEE for the composite index, which combines the three measurements of mite level, indicated that colonies inoculated with mites from both lightly and heavily managed backgrounds had significantly (Wald = 5.99, *P* = 0.014 and Wald = 4.55, *P* = 0.033, respectively) higher mite levels than the negative controls (Figure 1d). We did not find significant differences in mite levels within mite donor colony treatment groups.

### Colony strength and survival analysis

**Figure 2.**
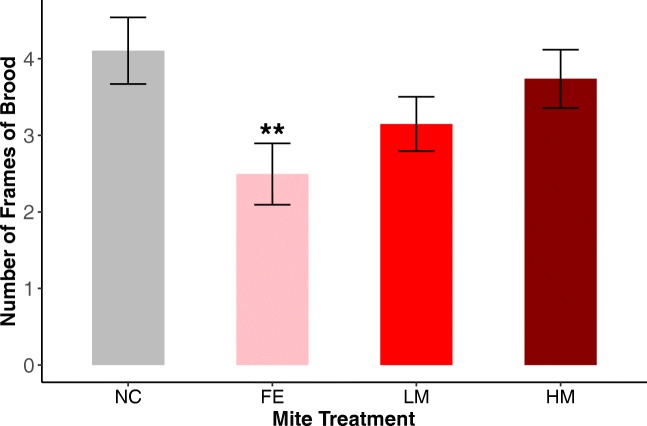
Number of frames of brood by treatment over the course of the experiment (NC = Negative Control, FE = Feral, LM = Lightly Managed, HM = Heavily Managed). A GEE model found significantly (Wald = 8.27, *P* = 0.0040) fewer frames of brood in the colonies inoculated with mites from a feral background. Note that the trend in the experimental treatment groups is opposite to what we predicted. Error bars represent SEM.

**Figure 3.**
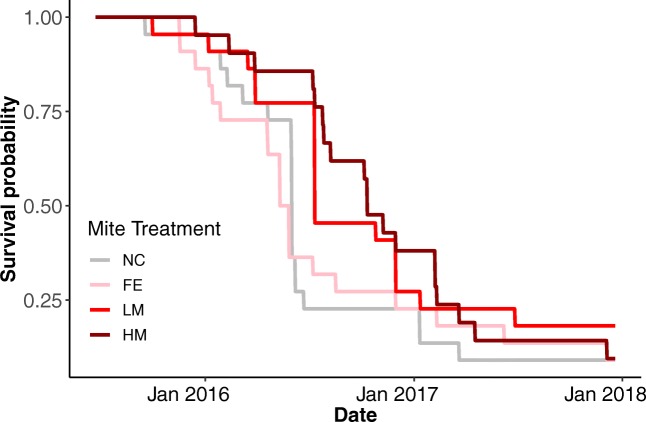
Survival curves by mite treatment (NC = Negative Control, FE = Feral, LM = Lightly Managed, HM = Heavily Managed). A Cox proportional hazard model with interval censoring did not find a significant difference between the groups.

The GEE model for the amount of brood showed that colonies inoculated with mites from feral backgrounds had significantly (Wald = 8.27, *P* = 0.0040) lower levels of brood production (Figure 2). The models for adult bee population and honey stores did not show any significant differences between the treatment groups and the negative control colonies. The feral and heavily managed treatments showed pairwise within treatment differences for adult bees based on mite donor colonies. The feral treatments had three significantly different pairwise comparisons (Wald = 19.67, *P* = 9.2 × 10^−6^ to Wald = 4.13, *P* = 0.042). The heavily managed treatments had five significantly different pairwise comparisons (Wald = 14.38, *P* = 0.00015 to Wald = 3.91, *P* = 0.048). Eighty-six percent (76 of 88) of the colonies died over the 2-year experiment. The Cox survival analysis did not show a significant difference in survival between the different treatment groups (Figure 3).

## Discussion

### Overview

The conditions for *V. destructor* are substantially different in managed bee colonies versus feral bee colonies (Seeley 67). The colony densities found in managed colonies far exceed those found in feral populations and may facilitate disease transmission (Seeley and Smith 68). According to theory, increased transmission between honey bee colonies may alter selection pressure to favor increased replication and virulence (Brosi et al. 15). We performed a large replicated study assessing how mites from different management backgrounds interacted with honey bees from a single background. We were able to replicate varroosis by standardizing bee background, clearing mites, and inoculating with controlled doses of mites in a large replicated study, which has not been documented before. Our work provides evidence consistent with theory that densities in managed colonies have favored *Varroa* destructor strains with increased growth rates. Specifically, we found increased levels of mites in colonies inoculated with mites taken from managed honey bee populations. However, we did not find the negative consequences we expected for colony strength and survival based on increased mite levels. In fact, for one response variable (brood production), we found that colonies inoculated with mites from feral backgrounds had a negative colony strength outcome relative to bees inoculated with mites from managed backgrounds.

### Mite infestation

Our finding of increased levels of *V. destructor* mites in colonies inoculated with mites from managed backgrounds (Figure 1) suggests that honey bee management conditions have favored increased mite reproductive rates. While these levels were not always significantly different from negative controls for each mite measure (Figure 1a–c), the trend was always consistent with our predictions, with colonies inoculated with mites from feral backgrounds exhibiting the lowest mite levels and mites from managed backgrounds showing increased mite burdens. The composite index of all three mite measures (Figure 1d) reduced within-group variation and showed that colonies inoculated with mites from managed backgrounds had increased levels of infestation. This is consistent with the idea that mites from feral vs managed backgrounds are under different selection pressures with potential differences in mite growth and/or virulence (Corrêa-Marques et al. 20, 21).

### Colony strength and survival analysis

We found significant within-treatment differences based on mite donor colony for adult bee population in apiaries inoculated with mites from feral or heavily managed bees. This indicates genetic variation in mites among feral and heavily managed bee populations, as has been found in other studies (Dynes et al. 29). While we did not find significant differences in adult bee population or honey stores across treatment groups, we found that bees inoculated with feral-background mites produced less brood than bees inoculated with mites from managed backgrounds (Figure 2). This was surprising because we expected the opposite: that higher levels of mites would lead to negative colony strength outcomes. There are five potential explanations for this pattern that we consider here.

First, the bees we used could be adapted to the mite strain that they coevolved with. Predicting the outcome of host-parasite interactions, such as in the honey bee—*V. destructor* system—can be complicated by interactions between host and parasite genotype. Genotype-by-genotype (G × G) interactions mean that some parasite strains are more successful against some hosts and some hosts less susceptible to certain parasite strains (Lambrechts et al. 45). When G × G interactions occur, no single parasite strain optimally infects all hosts, while no single host strain is optimally defended against all parasite strains (Carius et al. 18; Lambrechts et al. 45; de Roode and Altizer 24). Both theory and empirical studies indicate that coevolution can lead to increased host tolerance; as a consequence, a novel parasite strain from another evolutionary background can lead to more virulence than a coevolved parasite (Greischar and Koskella 35; Miller et al. 56; Read et al. 61; Hawley et al. 37; Gibson et al. 34). If this is the case, the observed patterns of mite growth and colony strength may be due to a genetic mismatch between lightly managed bees and mites from feral colonies, with lightly managed bees resisting, but not tolerating, mites from feral colonies. This means that the bees are able to keep parasite population levels in check (resistance) but are unable to cope with the damage caused by these lower levels of parasites (tolerance) (Restif and Koella 63; Best et al. 8). Thus, while we would predict that the higher transmission opportunities in managed honey bees select for greater mite virulence, we may also predict greater selection for host resistance and tolerance, and the existence of mismatches in coevolved mite and honey bee strains may make virulence outcomes more difficult to predict. A full cross-infection experiment using bees from different backgrounds (in addition to mites of different backgrounds, as we assessed here) is needed to follow up and explore this hypothesis.

Second, honey bee queens may adjust their egg laying frequency based on mite-induced bee mortality. This pattern of increased brood production as a potential means of compensation for higher brood parasitism in *V. destructor*-infested colonies was noted by Delaplane and Hood (26). Third, our negative controls, which were initially cleared of mites and not inoculated, had greater mite levels than we expected. This suggests that horizontal transmission of mites from outside the experiment could have occurred (Nolan and Delaplane 58). We isolated our experimental apiaries from all known colonies by at least 5 km to minimize this potential, but we cannot discount this as a possibility. Fourth, our mite clearance protocol may not have been as successful as we anticipated, and residual mite populations could have overtaken the inoculated population. However, our first sticky board samples taken after clearance and before inoculation showed most colonies having zero mites and an overall low average of 2.29 mites detected in the 72-h sample per colony. Thus, our inoculation of 100 mites should have overwhelmed any residual mite population. Finally, it is well known that the negative consequences of *Varroa* destructor infestation are both due to the mites themselves and the viruses they transmit, and differences in viral virulence are well established (Anderson 4; Vojvodic et al. 69; McMahon et al. 53). As such, it is possible that feral mites harbor different populations of viruses than those circulating in managed colonies and these feral viruses could have differential virulence or G × G interactions, leading to distinct health outcomes relative to mite infestation on their own in the absence of viruses.

Colony level mortality was a key measurement in our assessment of virulence of *Varroa destructor* on the honey bee colonies. The level of colony mortality (86%) across 2 years by the simple addition of mites indicates just how virulent *V*. *destructor* mites are for honey bee colonies. These findings are in line with another study that determined *V. destructor* was responsible for > 85% of the colony mortalities (Guzmán-Novoa et al. 36). However, we did not find an effect of mite background on colony survival (Figure 3). We had expected that the higher mite levels in colonies inoculated with mites from managed backgrounds would translate into worse health outcomes and reduced colony survival in these colonies. That we did not see these results suggests that there are other factors such as queen health (Amiri et al. 3) or viral infections that play a more important role than mite infestation. Additionally, the finding that our negative controls had similar survival outcomes as our treatment groups demonstrates that a single treatment for *Varroa destructor* infestations is ineffective, even when that treatment clears all or nearly all mites from a colony. One study found that while a single treatment of oxalic acid caused 97.6% mortality in *V. destructor* mites, an additional treatment resulted in 99.6% mortality leaving the possibility that a small population of mites could reestablish after a single treatment (Al Toufailia et al. 1).

### Future research

While our study provides insights into how mites from different backgrounds interact with bee colonies of a similar background, our results also indicate that a cross-infection study with bees from different backgrounds would help us further understand the trade-offs that occur in this system. Specifically, we suggest that future studies explore how human management contributes to virulence-transmission trade-offs by measuring transmission and virulence of mites introduced into mite-free apiaries such as Hawley et al. performed with a bird disease (2013). Additionally, we need to determine the conditions under which mite levels are dissociated from colony harm. Future work needs to focus on the role viruses play in the *Varroa destructor*-honey bee system. This three-way system could interact in potentially unexpected ways including mechanisms that confound our present understanding.

## Conclusion

Host population densities in managed honey bee apiaries are vastly different than what *Varroa destructor* experiences in feral honey bee populations. We provide evidence consistent with the idea that selection pressures on mites in these managed conditions favor increased reproductive rates. This could act to increase the transmission rate in these managed environments. However, we did not find negative strength and survival outcomes that we expected with these higher mite burdens. Mites from feral backgrounds may have caused negative health outcomes due to a mismatch in coevolved bee and mite strains. Future research needs to determine the conditions under which mite levels are dissociated from virulence and whether human management of bee colonies is driving selection for more damaging mites.

## Electronic supplementary material

ESM 1 (DOCX 574 kb)
