# Supplementary figures and images for: Assessing virulence of Varroa destructor mites from different honey bee management regimes
Source: Apidologie. 2019 Dec 10;51(2):276–89. doi: 10.1007/s13592-019-00716-6 (PMC7175645; doi:10.1007/s13592-019-00716-6)

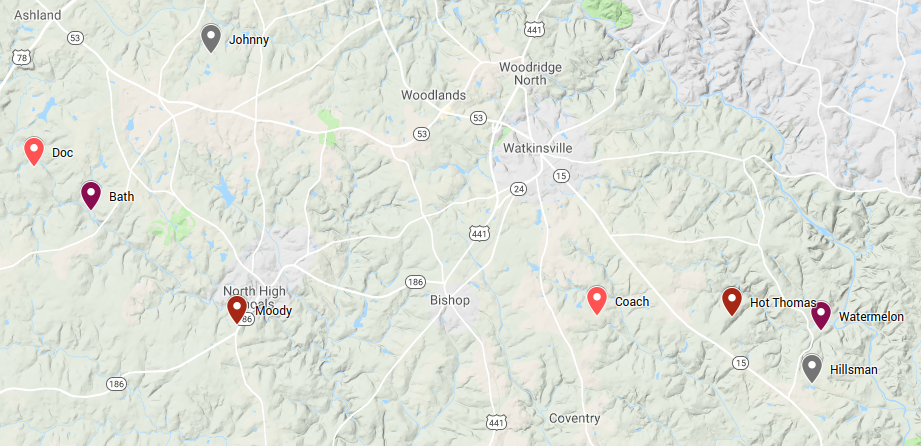


Figure S1: Map of apiary locations

Supplement: Supplementary file 1 — (DOCX 574 kb) [file 13592_2019_716_MOESM1_ESM.docx]
